# Platelet-Released Growth Factors Modulate the Secretion of Cytokines in Synoviocytes under Inflammatory Joint Disease

**DOI:** 10.1155/2017/1046438

**Published:** 2017-11-19

**Authors:** Mersedeh Tohidnezhad, Andreas Bayer, Biljana Rasuo, Jennifer Vanessa Phi Hock, Nisreen Kweider, Athanassios Fragoulis, Tolga Taha Sönmez, Holger Jahr, Thomas Pufe, Sebastian Lippross

**Affiliations:** ^1^Institute of Anatomy and Cell Biology, Medical Faculty, RWTH Aachen University, Wendlingweg 2, 52074 Aachen, Germany; ^2^Department of Heart and Vascular Surgery, University Hospital of Schleswig-Holstein, Kiel, Germany; ^3^Department of Oral, Cranio-Maxillofacial and Facial Plastic Surgery, Hospital Karlsruhe of University Freiburg, Karlsruhe, Germany; ^4^Frankfurt Orofacial Regenerative Medicine-Lab (FORM), University Hospital Frankfurt Goethe University, Frankfurt am Main, Germany; ^5^Department of Orthopaedic Surgery, RWTH Aachen University, Pauwelsstr. 30, 52074 Aachen, Germany; ^6^Department of Traumatology, University Hospital of Schleswig-Holstein, Kiel, Germany

## Abstract

The etiology and pathogenesis of rheumatoid arthritis (RA) are marked by a complex interplay of various cell populations and is mediated by different signaling pathways. Traditionally, therapies have primarily focused on pain relief, reducing inflammation and the recovery of joint function. More recently, however, researchers have discussed the therapeutic efficacy of autologous platelet-rich plasma (PRP). The main objective of this work is to examine the influences of platelet-released growth factor (PRGF) on human synoviocytes under inflammatory conditions. Additionally, it is checked to which extend treatment with platelet concentrate influences the release of cytokines form synoviocytes. For this purpose, an in vitro RA model was created by stimulating the cells with the TNF-*α*. The release of cytokines was measured by ELISA. The cytokine gene expression was analyzed by real-time PCR. It has been observed that the stimulation concentration of 10 ng/ml TNF-*α* resulted in a significantly increased endogenous secretion and gene expression of IL-6 and TNF-*α*. The anti-inflammatory effect of PRGF could be confirmed through significant reduction of TNF-*α* and IL-1*β*. An induced inflammatory condition seems to cause PRGF to inhibit the release of proinflammatory cytokines. Further study is required to understand the exact effect mechanism of PRGF on synoviocytes.

## 1. Introduction

Rheumatoid arthritis (RA) is a common chronic inflammatory disease that affects joints and is followed by swelling and pain. The impact of this inflammatory disease affects both the ability to perform in daily life activities and functional capacity [1].

Therapies of RA aim to reduce inflammation and pain and thus prevent joint and tissue damage. Nonsteroidal anti-inflammatory drugs (NSAIDs) and biological substances such as tumor necrosis factor-*α* (TNF-*α*) inhibitors are commonly used therapeutic applications.

Platelet concentrates (PC) are frequently used as an autologous injectable preparation for the treatment of various musculoskeletal pathologies [2–4]. The preparation is fairly simple and can be performed in an outpatient setting. The general rationale for clinical application of PC is its content of high concentrations of regenerative proteins that are naturally delivered by thrombocytes. In order to enhance and attenuate tissue regeneration, PC can be applied locally [5, 6].

The vast majority of disorders that are treated with platelet concentrate, that is, Achilles tendinitis, plantar fasciitis, and epicondylitis, have an inflammatory pathophysiology in common. In recent studies, the use of PC for arthritis has been discussed. Even though these findings have to be taken with care because they provide little evidence, a further investigation seems promising [5, 7, 8]. An in vitro study by Tong et al. shows that PRP inhibits the nuclear factor ĸ beta (NFĸB) and reduces the interleukin 1 beta (IL-1*β*) and TNF-*α* expression of synoviocytes after treatment with lipopolysaccharide (LPS) [5]. The quality of platelet concentrate is dependent on the donor as well as on preparation procedure [9–12]. Several studies showed that variation leads to inconsistent results [13]. We purport that the effectivity of PRGF varies between cell types and tissues. In previous studies, we could show that lower concentrations of PRGF are more effective in inducing tenogenic markers in tenocytes as an example for low vascularized tissue. Vice versa, high concentrations of PRGF were more advantageously for osteoblasts and bone tissue as an example for high vascularized tissue. The fundamental mode of action of thrombocytes and released growth factor of them on synoviocytes has not been sufficiently investigated so far. Therefore, the aim of this study was to further investigate the effect of PRGF, as a mixture of various cytokines and growth factors without leucocytes and plasma participation, on synoviocytes. We aimed also to investigate if addition of PRGF can regulate the endogenous expression of inflammatory mediators in synoviocytes. We hypothesize that plasma-free PRGF acts as an anti-inflammatory addition and prevents the TNF-*α*, IL-6, and IL-1*β* expression. In order to further elucidate possible actions of platelet concentrates in arthritis, we have investigated the effect of PRGF on an *in vitro* model for inflammatory arthritis using TNF-*α*-stimulated K4IM cells.

## 2. Materials and Methods

### 2.1. Preparation of PRGF

PRGF was produced from liquid-preserved platelet concentrates (PC) obtained by platelet apheresis in accordance with the current German ethics laws (EK116/10 local ethical board RWTH Aachen University). The PC of 9 × 10^9^ per ml were not older than one day and contained less than 5 × 10^4^ leukocytes. 2 ml PC was centrifuged at 2000*g* for 10 min, washed twice with citrate buffer, and then centrifuged again at 2000*g* for 10 min to remove the fibrinogen and other plasma components. The pellet was then resuspended in a total of 1 ml culture medium to achieve 8–10-fold concentration of platelet. Two cycles of freezing-thawing were used for the activation of platelets. Activated platelets were centrifuged at 18,000*g* for 1 min to remove cell debris. The collected supernatant is PRGF. PRGF in various concentrations was then added to the medium.

### 2.2. Cultivation of Human Synoviocytes

A stable human synoviocyte line (K4IM, a generous gift from Christian Kaps, Charite, Berlin, Germany) which is immortalized with the SV40 T antigen was used for in vitro study [14–17]. The cells were cultivated in monolayers in Dulbecco's phosphate-buffered saline (DMEM) (GIBCO®, Thermo Fisher Scientific) containing 10% fetal calf serum (FCS) and 1% penicillin-streptomycin. Cells were incubated at 37°C in 95% humidified air and 5% CO_2_ atmosphere. For subculture, cells were detached with 1% trypsin (GIBCO, Thermo Fisher Scientific) treatment. For life cell imaging and phase contrast microscopy, Keyence BZ-9000 microscope was used (Keyence, Japan).

### 2.3. Stimulation of Cells with Recombinant Human TNF-*α*

10^5^ cells/ml were seeded in a 6-well plate and then cultivated in culture medium for 24 h. The medium was replaced by serum-starved medium containing 1% FCS. Various concentrations of recombinant human TNF-*α* were added in culture media for 30 min and 1 h.

### 2.4. Treatment of Cells with PRGF

10^5^ cells/ml or 10^6^ cells/ml were seeded into fresh 6-well plates or 10 cm petri dishes, respectively, and cultivated for 24 h in synoviocyte medium to attach the cells. Then, the medium was replaced by serum-starved medium containing 1% FCS. Half of the well plate was stimulated with 10 ng/ml TNF-*α* for 30 min. Then PRGF in concentration of 0%, 5%, and 10% was added to the media of cells with and without TNF-*α* for 6 h.

### 2.5. ELISA

For ELISA analysis, after treatment of synoviocytes, the cells were washed twice with phosphate-buffered saline (PBS) and incubated for another 24 h in serum-starved medium to allow the release of cytokines into the supernatant. The supernatant aliquots (200 *μ*l) were used for ELISA. The total amount of protein was determined using BCA (bicinchoninic acid) kit (Pierce Chemical). The same protein concentrations were used, and the levels of cytokines were analyzed by sandwich ELISA (vascular endothelial growth factor (VEGF), IL-6, IL-1*β*, and TNF-*α*: R&D Systems, Minneapolis, MN, USA, IL-10: PeproTech, USA).

### 2.6. Real-Time RT-PCR

After cells were treated, the cells were washed twice with phosphate-buffered saline (PBS) and incubated for another 6 h in serum-starved medium. RNA was extracted with NucleoSpin RNA XS (Macherey Nagel, Germany) according to the manufacturer's protocol. The RNA concentration was determined by photometric analysis using the NanoDrop 1000 system (PEQLAB Biotechnologie GmbH). Real-time PCRs were processed in triplicate using the ABI StepOnePlus™ apparatus (Applied Biosystems) in a total volume of 15 *μ*l containing 70–100 ng of cDNA, gene-specific primers, and SYBR Green I reagent (Applied Biosystems). The target genes TNF-*α* (FW 5′-GGTCTTTGCCTTTTATCCCTCC-3′ and RV 5′-AAGCTCCCCCTCTTTTTCAGG-3′) (MWG, Germany), IL-1*β*, and IL-6 (Qiagen, Germantown, MD, USA) were analyzed. Beta-2-microglobulin (B2M) (FW 5′-TGCTGTCTCCATGTTTGATGTATCT-3′and RV 5′-TCTCTGCTCCCCACCTCTAAGT-3′) served as internal control.

### 2.7. CyQuant Cell Proliferation Assay

10^4^ cells were seeded into a fresh 96-well plate and cultivated for 24 h in order to attach the cells. The cells were then treated with various concentrations of PRGF for 24 h. The medium was removed and samples were frozen and stored at −70°C. After the cells were thawed, 200 *μ*l CyQuant GR dye/cell lysis buffer was added to each well in accordance with the manufacturer (Thermo Fisher Scientific, USA). The DNA content was measured using a fluorescence microplate reader (Infinite M200, TECAN) at 480 nm excitation and 520 nm emission and related to a standard number of cells (counted with trypsinized cells).

### 2.8. Cell Titer-Blue® Cell Viability Assay

To evaluate the optimal minimal concentration of FCS to assembling the serum-starved media, a Cell Titer-Blue cell viability assay was performed. 10^4^ cells were seeded into a fresh 96-well plate and cultivated with synoviocyte media. After 24 h, the medium was replaced by serum-starved media containing various concentrations of FCS. The samples were incubated for another 24 H. Media were supplemented with 60 *μ*l CTB reagent (1 : 5 diluted in serum-staved media). The cells were incubated for 2 h. Fluorescence was detected at 560 nm excitation and 590 nm emission using fluorescence microplate reader (Infinite M200, TECAN). The lowest concentration of FCS, which had no significant effect on cell viability, was chosen as serum-starved media.

### 2.9. Statistical Analysis

For the analysis of PRGF effects on synoviocyte viability and proliferation, 6 various PRGF concentrates from various patients were used. The assay was running in duplicate each and mean values were used for statistical analysis and were compared using a one-way ANOVA and nonparametric Kruskal-Wallis test. Results were expressed as the mean ± standard error (SEM). For ELISA experiments, 6–9 various PRGF concentrates from various patients were used.

The illustrated results in group diagrams were analyzed using a two-way ANOVA, nonparametric, multiple comparisons. Differences were considered as significant with values of *p* < 0.05. All statistical graphs and analyses were created with GraphPad Prism 6.0 (GraphPad Software, La Jolla, CA, USA).

## 3. Results

### 3.1. Selection of Optimal Serum-Starved Medium

**Figure 1:**
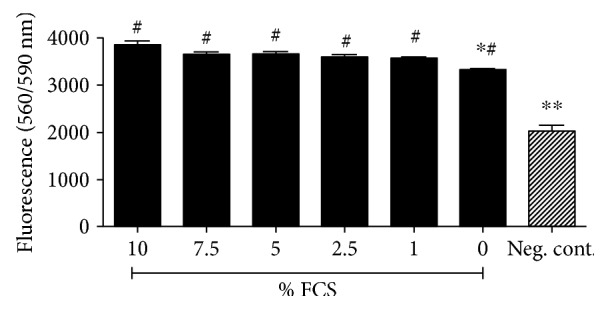
Selection of optimal serum-starved medium. To find the optimal serum-starved medium, K4IM cells were cultured with various concentrations of FCS for 24 hours. Cell viability was measured using CTB cell viability assay. 1% FCS shows no reduction of cell viability and toxicity and was chosen as a serum-starved medium. ^∗^Significant versus the control group with 10% PRGF. ^#^Significant versus the negative control, *p* ≤ 0.05.

To avoid cell proliferation during experiments, serum-starved media were used. To confirm an appropriate serum-starved medium, synoviocytes were cultured with concentrations ranging from zero to ten percent FCS. The cell viability was evaluated using a Cell Titer-Blue (CTB) cell viability assay (Figure 1). 1% FCS showed no reduction of cell viability (35,629 ± 192.0 compared to the control 38,482 ± 836.6) and no toxicity (compared to negative control 20,178 ± 1316) and was adapted as serum-starved medium (*n* = 6, ^∗^*p* ≤ 0.05 and ^∗∗^*p* ≤ 0.01).

### 3.2. In Vitro Model for Inflammatory

**Figure 2:**
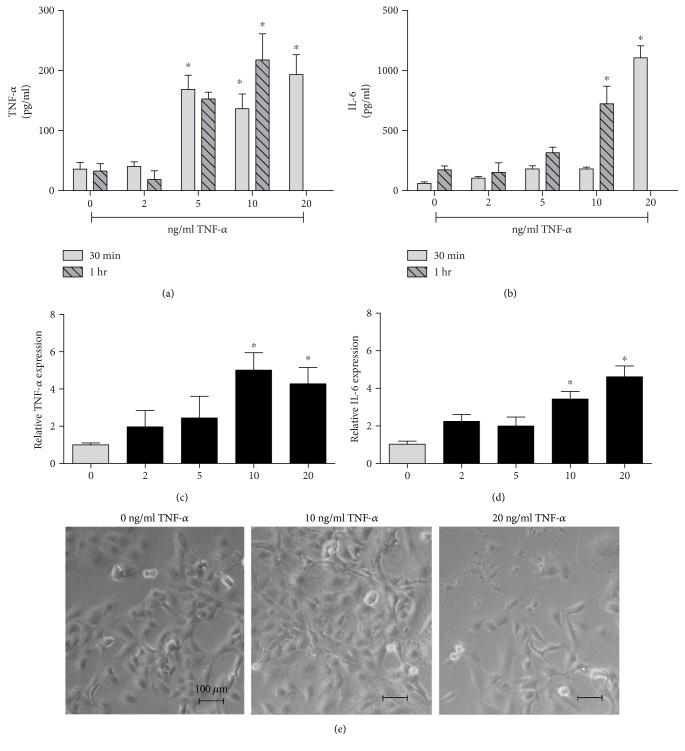
*In vitro* model for inflammatory. K4IM was pretreated with 2, 5, 10, and 20 ng/ml TNF-*α* for one hour and 30 minutes. (a) Using 10 ng/ml TNF-*α*, the release of endogenous TNF-*α* is significantly higher than the control group at both 30 min and 60 minutes. (b) The release of IL-6 is also significantly higher than the control group after 30 and 60 minutes. For in vitro inflammatory model, cells were pretreated with 10 ng/ml TNF-*α* for 30 minutes. The ELISA results were confirmed by gene expression analysis where 10 ng/ml TNF-*α* led to a peak relative TNF-*α* expression (c) and an increase in IL-6 expression (d), respectively. When a concentration of 20 ng/ml of TNF-*α* was applied, the cell morphology deteriorated dramatically and apoptosis was induced after 60 min (e). ^∗^Significant versus the control group without TNF-*α*, *p* ≤ 0.05.

First, we undertook experiments to characterize an in vitro model for inflammatory arthritis by stimulating K4IM with TNF-*α* (Figures 2(a)–2(d)). K4IM were pretreated with different concentrations of TNF-*α* (2, 5, 10, and 20 ng/ml) for one hour and 30 minutes. After cell treatment, the cells were washed and further incubated for another 24 h in serum-starved medium for cytokine release. Using 10 ng/ml TNF-*α*, the release of endogenous TNF-*α* was 136.3 ± 24.5 pg/ml and 217.6 ± 43.7 pg/ml. After 30 and 60 minutes, the release of IL-6 was 180.0 ± 15.8 pg/ml and 722.8 ± 147.2 pg/ml after 30 and 60 minutes, respectively, *n* ≥ 5, *p* ≤ 0.05. When a concentration of 20 ng/ml of TNF-*α* was applied, the cell morphology deteriorated dramatically after 30 min and apoptosis was induced after 60 min (Figure 2(e)). Therefore, 20 ng/ml is considered as an overdose and is not used for the study. For verification of ELISA data, real-time RT-PCRs were performed. The cells were treated with various concentration of TNF-*α* for 30 min, washed, and further incubated in serum-starved medium for another 6 h. The ELISA results were confirmed by gene expression analysis where 10 ng/ml TNF-*α* led to a peak relative TNF-*α* expression of 5.0 ± 0.9 and an increase in IL-6 expression of 3.4 ± 0.4 after 6 hours, respectively, *n* = 8, *p* ≤ 0.05.

### 3.3. Effects of PRGF on Synoviocyte Viability and Proliferation

**Figure 3:**
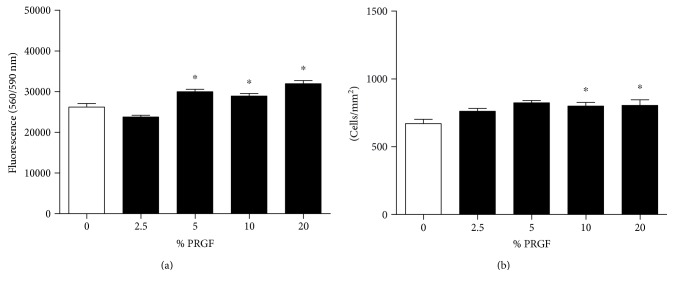
Effects of PRGF on synoviocyte viability and proliferation. The influence of PRGF on K4IM cell viability was determined by CTB cell viability assay (a). The addition of 5%, 10%, and 20% PRGF increase significantly the cell viability after 24 hours. The influence of PRGF on K4IM cell proliferation was determined by CyQuant cell proliferation assay (b). Different concentrations of PRGF induce the higher proliferation rate in K4IM cells compared to the group without PRGF. ^∗^Significant versus the control group without PRGF, *p* ≤ 0.05.

Next, we studied the influence of PRGF on K4IM cells (Figures 3(a) and 3(b)). The addition of 5%, 10%, and 20% PRGF positively influences the cell viability in a CTB cell viability assay after 24 hours (29,977 ± 612.7, 28,926 ± 617.0, and 31,938 ± 773.6 560_Ex_/590_Em_, resp.) when compared to the control group without PRGF (26,188 ± 900.9 560_Ex_/590_Em_) *n* = 6, *p* ≤ 0.05. 5%, 10%, and 20% PRGF induced the proliferation of K4IM cells in a CyQuant cell proliferation assay (5%: 824.6 ± 16.1 cells/cm^2^, 10%: 799.3 ± 27.8 cells/cm^2^, and 20%: 805.1 ± 41.5 cells/cm^2^ compared to the control: 670.7 ± 31.4), *n* = 6, *p* ≤ 0.05.

### 3.4. Effects of PRGF on TNF-*α* Pretreated Synoviocyte Cytokine Release and Cytokine Expression

#### 3.4.1. Release of Endogenous TNF-*α* from the Cells after TNF-*α* Stimulation and PRGF Treatment (Figure 4(a))

Treatment with 5 or 10% of PRGF showed no effect on endogenous TNF-*α* release (gray column, 375.0 ± 40.48 and 315.8 ± 42.46, respectively, compared to 483.0 ± 28.8; *n* = 9, ^∗^*p* ≤ 0.05 and ^∗∗^*p* ≤ 0.01). Addition of TNF-*α* to the cell culture media (+TNF-*α*, stripped column) led to significant increase of endogenous TNF-*α* release from the cells (compare of gray and stripped column by 0% PRGF).

**Figure 4:**
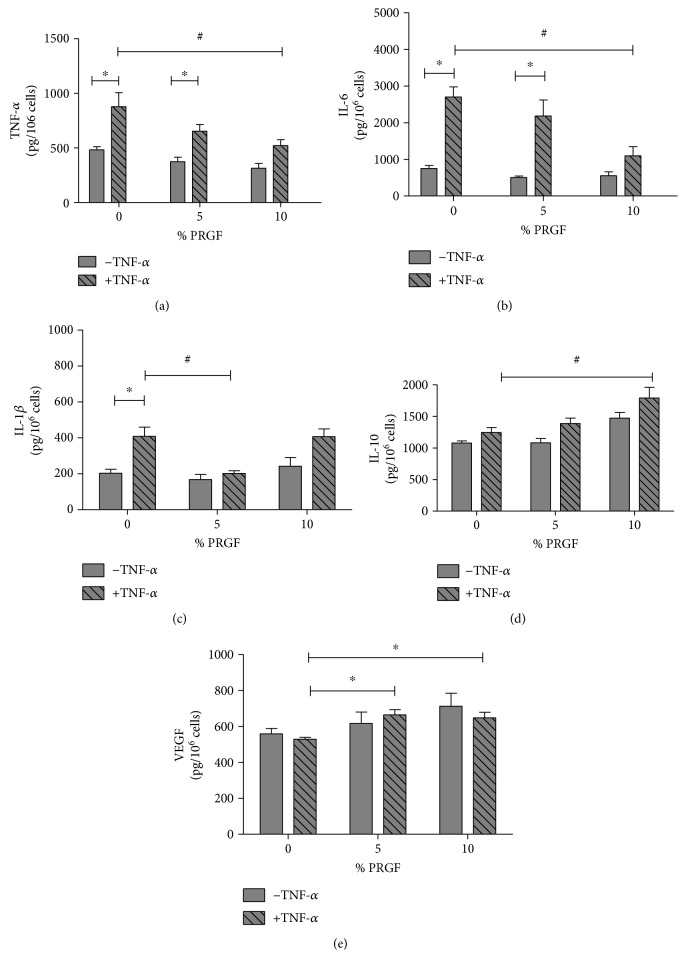
Effects of PRGF on TNF-*α* pretreated synoviocyte cytokine release. 5% and 10% PRGF was added to TNF-*α* pretreated K4IM media for 6 h. After twice wash step with PBS, the medium was replaced by serum-starved media for 24 h. The release of endogenous cytokines was measured by ELISA. (a) Addition of 10% PRGF to the media of TNF-*α* pretreated (+TNF-*α*) K4IM and untreated cells led to significant reduction of endogenous TNF-*α* release. (b) IL-6 protein level is reduced in +TNF-*α* K4IM at a PRGF concentration of 10%. PRGF has no effect on IL-6 release in untreated cells. (c) The endogenous release of IL-1*β* is reduced when 5% PRGF were added to prestimulated K4IM. 10% PRGF has no effect on IL-1*β* release in untreated or prestimulated cells. (d) 10% PRGF in the medium of both untreated K4IM and TNF-*α* prestimulated K4IM increases the IL-10 protein release in media. (e) 5% and 10% PRGF in the medium of TNF-*α* prestimulated K4IM resulted in an increased concentration of VEGF, *n* = 9. ^∗^Significant versus the control group without TNF-*α* prestimulation and without PRGF. ^#^Significant versus group with TNF-*α* and without PRGF, *p* ≤ 0.05.

Addition of 10% PRGF to the media of TNF-*α* pretreated K4IM (+TNF-*α*) led to significant reduction of endogenous TNF-*α* release (489.6 ± 47.6 compared to the 877.9 ± 128.6) up to the level of untreated cells (Figure 4(a), stripped column).

#### 3.4.2. Release of IL-6 from the Cells after TNF-*α* Stimulation and PRGF Treatment (Figure 4(b))

Addition of both 5% and 10% PRGF had no effect on endogenous IL-6 release in untreated cells (gray column). The release of IL-6 was reduced in +TNF-*α* K4IM at a PRGF concentration of 10% (1129 ± 286.0 compared to 2701 ± 307.1). PRGF has no effect on IL-6 release in untreated cells, *n* = 9, *p* ≤ 0.05 (Figure 4(b)).

#### 3.4.3. Release of IL-1*β* from the Cells after TNF-*α* Stimulation and PRGF Treatment (Figure 4(c))

Addition of both 5% and 10% PRGF had no effect on endogenous IL-1*β* release in untreated cells (gray column). The endogenous release of IL-1*β* was reduced when 5% PRGF were added to prestimulate K4IM (202.0 ± 15.2 compared to 408.7 ± 50.57). 10% PRGF has no effect on IL-1*β* release in untreated or pretreated cells, *n* = 6, ^∗^*p* ≤ 0.05.

#### 3.4.4. Release of IL-10 from the Cells after TNF-*α* Stimulation and PRGF Treatment (Figure 4(d))

Addition of 5% PRGF or pretreatment of cells with TNF-*α* had no effect on endogenous IL-10 release in untreated cells (gray column and stripped column, resp.).

Addition of 10% PRGF in the medium of K4IM led to significant release of anti-inflammatory cytokine IL-10 in both untreated cells (−TNF-*α*) and TNF-*α* pretreated (+TNF-*α*) cells (−TNF-*α*: 271.2 ± 38.7 compared to 205.7 ± 15.7 and +TNF-*α*: 407.5 ± 42.5 compared to 408.7 ± 50.6), *n* = 6, *p* ≤ 0.05 (Figure 4(d)).

#### 3.4.5. Release of VEGF from the Cells after TNF-*α* Stimulation and PRGF Treatment (Figure 4(e))

The release of endogenous VEGF was also investigated using ELISA. Pretreatment of cells with TNF-*α* had no effect on release of L-10 in K4IM cells.

Addition of PRGF has no significant effect on VEGF release of untreated cells, whereas addition of 5% and 10% PRGF led in TNF-*α* pretreated cells to an increase of VEGF release (664.2 ± 37.13 and 647.6 ± 42.3 compared to 529.0 ± 14.4, resp.), *n* = 6, *p* ≤ 0.05 (Figure 4(e)).

To confirm the effect of PRGF on inflammatory mediators, real-time RT-PCR was performed.

**Figure 5:**
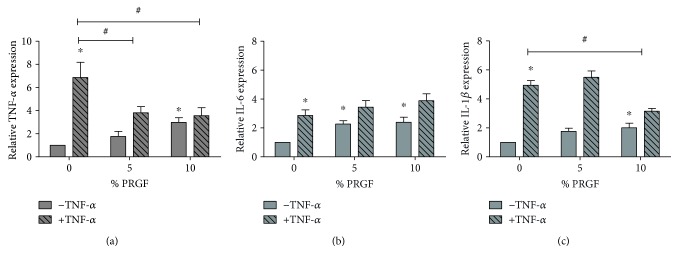
Effects of PRGF on TNF-*α* pretreated synoviocyte cytokine expression. To confirm the effect of PRGF on gene expression of inflammatory mediators, real-time RT-PCR was performed. (a) The endogenous expression of TNF-*α* is reduced when 5% and 10% PRGF are added to the medium. (b) Addition of 10% PRGF increases the IL-6 gene expression in untreated cells but has no significant effect on TNF-*α* prestimulated cells. (c) IL-1*β* gene expression is attenuated when 10% PRGF was added, *n* = 8. ^∗^Significant versus the control group without TNF-*α* prestimulation and without PRGF. ^#^Significant versus group with TNF-*α* and without PRGF, *p* ≤ 0.05.

The endogenous gene expression of TNF-*α* was reduced when 5% and 10% PRGF are added to the medium (3.8 ± 0.5 and 3.7 ± 0.6 compared to 6.9 ± 1.2, resp.). Addition of PRGF had no effect on endogenous TNF-*α* gene expression, *n* = 8, *p* ≤ 0.05 (Figure 5(a)). Addition of 10% PRGF increased the IL-6 gene expression up to 2.4 ± 0.4-fold of control group, *n* = 8, *p* ≤ 0.05 (Figure 5(c)). There was no effect on +TNF-*α* group if 5% or 10% PRGF was added in the media in comparison to the +TNF-*α* group without PRGF (3.4 ± 0.5 and 3.9 ± 0.6 compared to 2.9 ± 0.4, resp.), *n* = 8, *p* ≤ 0.05 (Figure 5(b)).

IL-1*β* gene expression was downregulated, when 10% PRGF was added (3.1 ± 0.2 compared to 4.9 ± 0.4). There was no significant effect between IL1-*β* gene expression on +TNF-*α* and −TNF-*α* group when 10% PRGF was added, *n* = 8, *p* ≤ 0.05 (Figure 5(c)).

## 4. Discussion

Synovitis is the characteristic reaction of the joint if its homeostasis is disturbed [18, 19]. Regardless of the cause, the endogenous release of TNF-*α* and IL-6 is usually increased whereas IL-10 and VEGF are reduced [20, 21].

Most studies focus on osteoarthritis of the knee joint and report favorable outcomes for intra-articular injections of platelet concentrate and its derivate in the knee joint [7, 22, 23] whereas others report beneficial effects in basal thumb joint arthritis [24].

TNF-*α* as an inductor of arthritis has long been recognized to play a key role in all types of arthritis which makes our model an appropriate laboratory setup to test the possible effects of PRGF.

In this study, we tested the anti-inflammatory potential of PRGF in vitro and investigated the modulation of growth factor releases from thrombocytes through cytokine release of synoviocytes in an *in vitro* model for arthritis.

PRGF, a derivate of PRP, with minimized content of plasma, fibrin, fibrinogen, and cell debris was used as a medium supplement in this study. This preparation has some advantages over conventional PRP. PRGF allowed us to analyze the role of sole platelets, without serum or plasma protein on the cells. There are significant differences observed between growth factors and cytokine levels in platelet concentrate when compared to the plasma and serum [10, 25]. We were able to show in previous work that the level of proinflammatory cytokines IL-6 and TNF-*α* in platelet concentrate is significantly less than in plasma and serum [25], whereas the level of some growth factors such as platelet-derived growth factor-BB (PDGF-BB), transforming growth factor (TGF-*β*), and VEGF are significantly higher than both plasma and serum [10, 25]. Furthermore, by using PRGF, the clotting of serum and medium components caused by fibrin formation in in vitro studies is prevented.

It is most likely that the huge number of well-investigated components of PRGF and other platelet concentrations can boost the expansion, that is, the viability and proliferation of many cell types including chondrocytes, synoviocytes, and tenocytes [6, 9, 26]. Therefore, these results comply with the obtained results from other studies.

We first implemented an in vitro model for inflammatory arthritis according to previously reported results. We confirmed that 10 ng/ml TNF-*α* represents an appropriate concentration to induce the production and expression of TNF-*α* and IL-6 that are typical for a synovial response to early joint inflammation [27–30].

The effect of sole cytokines on synoviocytes was somewhat expected as comparable results were published by various authors in the past [16].

When PRGF was added to the media of TNF-*α*-stimulated synoviocytes, we found a modulation of cytokine release that is suggestive of an anti-inflammatory effect. The TNF-*α*, IL-6, and IL-1*β* levels were significantly decreased compared to unstimulated cell cultures whereas IL-10 and VEGF release was increased.

PRGF contains a high variability of cytokines and growth factors such as TGF-*β* and BMPs, which inhibit the expression of proinflammatory cytokines in rheumatoid arthritis [30]. Further, PRGF contains a small amount of proinflammatory cytokines. The efficacy of these mediators at 5% PRGF can be limited by the presence of their specific receptors on the cell surface. The increase of PRGF concentrations up to 10% enhanced the proinflammatory cytokine supply in the media and should be critically reflected [25].

The increase in VEGF release could be interpreted as a hint for possible therapeutic use in rheumatoid arthritis. VEGF is a target of antirheumatic drugs and is accepted as an important factor within the pathomechanism of immunogenic arthritis [31]. The modulation by PRGF could therefore possibly be used to attenuate synovitis in rheumatoid arthritis in concert with the other effects we describe.

Proinflammatory effects of TNF-*α*, IL-6, and IL-1*β* have been described by many authors before, and hence this finding is plausible when seen in the context of platelet-released factors and synovitis [20, 21]. Our findings seem of particular interest if arthritis and the underlying pathology is well understood.

The data showed that PRGF has antiarthritic and immune regulatory effects in an in vitro and in vivo model of RA.

The model we used here acts as a simplified model for initial inflammatory state of synoviocytes and is limited by several points. This model is well established in our laboratory [29] and was used for some publications in our group and by other colleagues [32]. They used the K4IM cells, which are a stable human synoviocyte cell line obtained from healthy donors. These results should be confirmed using primary cells obtained from rheumatoid arthritis patients or human rheumatoid fibroblast-like cells such as MH7A or HSE [33–35]. Furthermore, in the complex processes of rheumatoid arthritis, there is a wide range of cells (T-cells, macrophages, chondrocytes, etc.), mediators, receptors, and pathways involved in the pathogenesis, which are not considered in our model.

## 5. Conclusion

In summary, we have implemented a cell-based system that allows for the investigation of a platelet concentrate (PRGF) that is implemented in the current clinical practice. We demonstrate a proliferative effect in cell cultures of K4IM cells and show that the pattern of modulation of endogenous release of important cytokines and VEGF is indicative for an overall anti-inflammatory action. Further investigations specifically identifying the underlying mechanisms of single factors in PRGF will be necessary to further understand the rationale behind the clinical application in osteoarthritis and rheumatologic disease.

## Abbreviations

°C: Degree Celsius
CTB: Cell Titer-Blue cell viability assay
DMEM: Dulbecco's phosphate-buffered saline
FCS: Fetal calf serum
FGF: Fibroblast growth factor
G: Gravitational
h: Hour
H: Human
IGF: Insulin-like growth factor
IL: Interleukin
LPS: Lipopolysaccharide
min: Minute
MMP: Matrix metalloproteinase
MSC: Mesenchymal stem cells
NFĸB: Nuclear factor ĸ beta
NSAID: Nonsteroidal anti-inflammatory drugs
OA: Osteoarthritis
PBS: Phosphate-buffered saline
PC: Platelet concentrates
PDGF-BB: Platelet-derived growth factor-BB
PMC: Platelet mediator concentrate
PRF: Platelet-rich fibrin
PRGF: Platelet-released growth factor
PRP: Platelet-rich plasma
TGF-*β*: Transforming growth factor beta
TNF: Tumor necrosis factor
VEGF: Vascular endothelial growth factor.
